# Iron homeostasis and tumorigenesis: molecular mechanisms and therapeutic opportunities

**DOI:** 10.1007/s13238-014-0119-z

**Published:** 2014-12-06

**Authors:** Caiguo Zhang, Fan Zhang

**Affiliations:** 1 Department of Biochemistry and Molecular Genetics, University of Colorado School of Medicine, Aurora, CO 80045 USA; 2 Orthopedics Department, Changhai Hospital Affiliated to Second Military Medical University, Shanghai, 200433 China

**Keywords:** Iron tumorigenesis, p53, Wnt, DNA repair, cell cycle

## Abstract

Excess iron is tightly associated with tumorigenesis in multiple human cancer types through a variety of mechanisms including catalyzing the formation of mutagenic hydroxyl radicals, regulating DNA replication, repair and cell cycle progression, affecting signal transduction in cancer cells, and acting as an essential nutrient for proliferating tumor cells. Thus, multiple therapeutic strategies based on iron deprivation have been developed in cancer therapy. During the past few years, our understanding of genetic association and molecular mechanisms between iron and tumorigenesis has expanded enormously. In this review, we briefly summarize iron homeostasis in mammals, and discuss recent progresses in understanding the aberrant iron metabolism in numerous cancer types, with a focus on studies revealing altered signal transduction in cancer cells.

## Introduction: overview of iron and cancer

**Figure 1:**
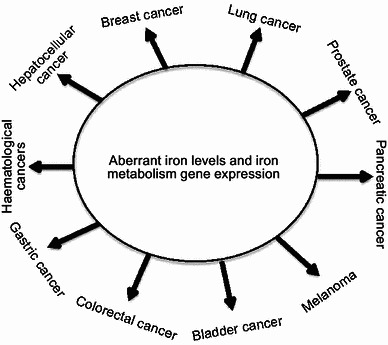
**Iron is implicated in multiple cancer types**. These cancers mainly include breast cancer, lung cancer, prostate cancer, pancreatic cancer, melanoma, bladder cancer, hepatocellular cancer, colorectal cancer, gastric cancer and haematological cancers

Iron serves important functions for mammalian cells as it is involved in cell proliferation, metabolism and growth (Torti and Torti, 2013). These processes are controlled by a variety of iron- and heme-containing proteins, including enzymes involved in DNA stability and cell cycle progression, mitochondrial enzymes involved in respiratory complexes, and detoxifying enzymes such as peroxidase and catalase (Torti and Torti, 2013; Zhang, 2014). Within the human body, iron biologically exists in two oxidation states: ferrous iron (Fe^2+^) and ferric iron (Fe^3+^) (Pantopoulos et al., 2012; Rouault, 2003). Iron has the property of gaining and losing electrons, which enables it to participate in Fenton reaction (Pantopoulos et al., 2012; Torti and Torti, 2013), in which Fe^2+^ donates an electron in a reaction with hydrogen peroxide (H_2_O_2_) to produce the hydroxyl radical (^•^HO) (Thomas et al., 2009), a reactive oxygen species (ROS). Human body needs to maintain systemic and cellular iron homeostasis by regulating iron acquisition, storage and efflux (Zhang, 2014). Iron homeostasis is not only required for iron-containing protein functions, but also critical for signal transduction and cellular microenvironment (Xiong et al., 2014). The elevated iron may result in the generation of ROS, which can damage lipids, proteins and DNA, eventually leading to tumorigenesis (Orrenius et al., 2011; Romero et al., 2014). It has been reported that numerous types of cancers are implicated by iron, such as lung cancer, breast cancer, prostate cancer, colorectal cancer, hepatocellular cancer, pancreatic cancer and hematological malignancies (Fig. 1) (Torti and Torti, 2013). On the other hand, iron deficiency caused anemia is one of the major public health problems, particularly in children and pregnant women (Denic and Agarwal, 2007; Miller, 2013). The recent studies also indicate that many patients with cancer have anemia (Munoz et al., 2014), but the cause is still to be determined.

Previous studies suggest that iron may function in tumour initiation, tumour growth, tumour microenvironment and metastasis (Mantovani et al., 2008; Sica et al., 2008). In cancer cells, pathways involved in iron acquisition, trafficking, storage and regulation are all perturbed, suggesting that iron metabolism is important for tumour cell survival (Torti and Torti, 2013). Additionally, iron can also contribute to DNA replication and repair processes, as well as cell cycle control in cancer cells (Torti and Torti, 2013; Zhang, 2014). Signalling through p53, Wnt, hypoxia-inducible factor (HIF), DNA replication, repair and cell cycle progression pathways may associate with altered iron metabolism in cancer (Torti and Torti, 2013). Thus, decreasing cellular iron levels, targeting iron metabolic pathways and iron-containing proteins may provide new tools for cancer therapy.

## Iron metabolism in mammals

Mammalian organisms have evolved sophisticated mechanisms to regulate systemic and cellular iron balance (Andrews and Schmidt, 2007; Pantopoulos et al., 2012).

### Systemic iron metabolism

Generally, systemic iron regulatory processes include several critical steps: (1) duodenal enterocytes acquire dietary iron via divalentmetal transporter 1 (DMT1), also known as solute carrier family 11 member 2 (SLC11A2), natural resistance-associated macrophage protein 2 (NRAMP2), or divalent cation transporter (DCT1) (Pantopoulos et al., 2012). DMT1 localizes on the apical surface and functions dependently on the reduction of Fe^3+^ to Fe^2+^ by duodenal cytochrome b (DcytB) (Pantopoulos et al., 2012); (2) spleenic reticuloendothelial macrophages are responsible for iron recycling from senescent red blood cells (Pantopoulos et al., 2012); (3) iron exporter ferroportin (Fpn) releases iron oxidized prior by hephaestin from Fe^2+^ to Fe^3+^ (Pantopoulos et al., 2012); (4) transferrin (Tf) located on plasma membrane acquires and delivers iron in the body (Pantopoulos et al., 2012); and (5) hepatic hormone hepcidin controls systemic iron trafficking and iron efflux from cells by regulating Fpn stability (Pantopoulos et al., 2012; Zhang, 2014).

### Cellular iron metabolism

**Figure 2:**
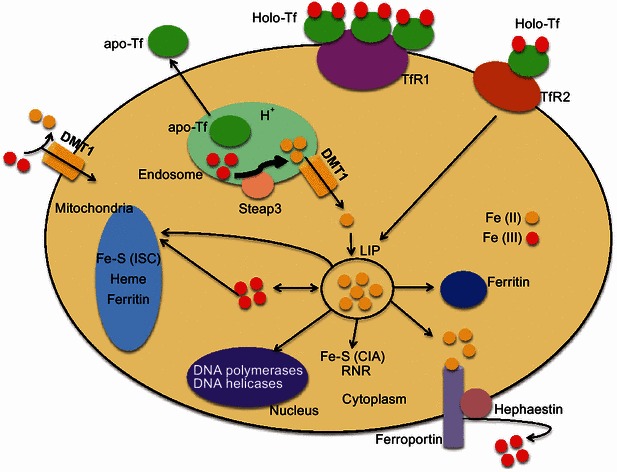
**Cellular iron metabolism in mammals**. Apo-Tf binds ferric iron to form holo-Tf. Holo-Tf further forms a complex with TfR1 on the cell surface and the complex undergoes endocytosis. Acidifying by a proton pump, ferric iron is released from holo-Tf in the endosome, where Steap3 reduces ferric iron to ferrous iron. Further, ferrous iron is transported across the endosomal membrane to the cytosol by DMT1. DMT1 also facilitates dietary ferrous iron absorption in the plasma. The released apo-Tf is recycled back to the plasma membrane to repeat another cycle. Newly acquired iron enters into cytosolic “labile iron pool” (LIP) (Pantopoulos et al., 2012). The LIP is utilized by iron-sulfur clusters (Fe-S) proteins, hemoproteins, RNR and other iron-containing proteins, which localize in different cellular compartments (Zhang, 2014). Cellular iron that is not utilized is either stored in ferritin or exported via ferroportin (Pantopoulos et al., 2012)

Cellular iron homeostasis is controlled by iron uptake at the plasma membrane, eliciting balanced iron distributions among cellular compartments and iron export (Valerio, 2007; Zhang, 2014). Briefly, most mammalian cells acquire iron via Tf to form holo-Tf (Anderson and Vulpe, 2009; Dunn et al., 2007), which further binds to transferrin receptor 1 (TfR1) to form holo-Tf-TfR1 complex on the iron-consuming cell membrane (Zhang, 2014). This complex is subsequently internalized by receptor-mediated endocytosis (Lill et al., 2012) and acidified in the endosome, facilitating the release of Fe^3+^ from holo-Tf (Zhao et al., 2010). The six-transmembrane epithelial antigen of the prostate 3 (Steap3) reduces Fe^3+^ to Fe^2+^, followed by transporting Fe^2+^ into the cytoplasm by DMT1 or transient receptor potential protein (TRPML1) (Zhang et al., 2012). Later, the holo-Tf-TfR1 complex disassembles and apo-Tf recycles back to the cell membrane to repeat another cycle (Pantopoulos et al., 2012). Thereafter, the newly acquired iron stores into the cytosolic “labile iron pool” (Gkouvatsos et al., 2012; Pantopoulos et al., 2012). The excess cellular iron is either stored in ferritin or exported via Fpn (Pantopoulos et al., 2012) (Fig. 2). Moreover, two iron regulatory proteins, namely, IRP1 and IRP2, can post-transcriptionally regulate cellular iron homeostasis (Zhang, 2014). In low iron condition, IRP1 and IRP2 proteins specifically bind to iron-responsive elements (IRE) in 3′- or 5′-UTR of the mRNA transcripts in TfR1, ferritin heavy (H) chain, ferritin light (L) chain, or DMT1 (Zhang, 2014). The IRE-IRP system functions importantly in the control of mammalian iron homeostasis (Pantopoulos et al., 2012). Consequently, these iron regulatory proteins are protected from degradation or their translations are inhibited (Anderson and Vulpe, 2009; Dunn et al., 2007; Kaplan and Kaplan, 2009; Muckenthaler et al., 2008).

## Iron is implicated in a variety of cancer types

**Figure 3:**
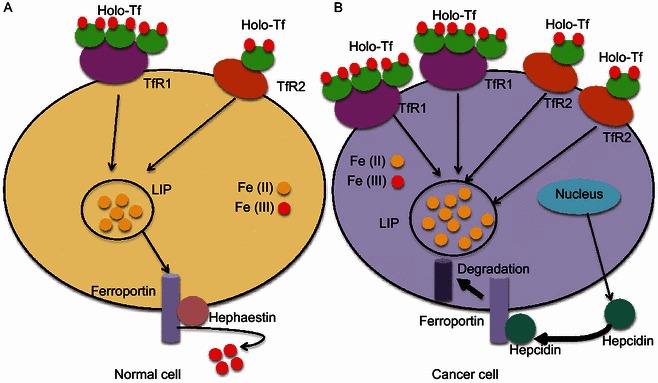
**Iron metabolism in normal cell and cancer cell**. (A) The expression of *Tf*, *TfR1*, *TfR2* and *hepcidin* is low, whereas the expression of iron exporter gene *FPN* is high in normal cells, leading to a small pool of labile iron (Torti and Torti, 2013). (B) Cancer cells exhibit increased expression of *TfR1* and *hepcidin*, but low levels of *FPN*, leading to an increased labile iron pool (Torti and Torti, 2013)

Multiple cancer types have been widely reported to exhibit abnormal iron contents or deficiency in iron uptake, utilization and storage (Fig. 3). These cancers mainly include lung cancer, breast cancer, prostate cancer, colorectal cancer, hepatocellular cancer, pancreatic cancer, haematological cancers, renal cell carcinoma and melanoma (Fig. 1) (Torti and Torti, 2013).

### Lung cancer

Lung cancers are generally categorized as small cell lung cancer (SCLC) and non-small cell lung cancer (NSCLC) (Vescio et al., 1990). During the past few years, hepcidin and several iron metabolism related proteins have been demonstrated to associate with lung cancer genesis and tumor cell proliferation (Xiong et al., 2014). Hepcidin expression is increased in tumor tissue and serum of NSCLC patients, and the increased serum hepcidin level is associated with lymph node metastasis and tumor clinical stage of NSCLC (Xiong et al., 2014). Iron related proteins, such as TfR1, H and L subunits of ferritin protein, also exhibit increased levels in lung cancer. In H1299 lung cancer cells, the induction of p53 decreases iron regulatory protein binding, leading to an increase in both H and L subunits of ferritin protein, but a decline of TfR1 level (Zhang et al., 2008). However, some studies reported elevated expression of *TfR1* in NSCLC patients (Kukulj et al., 2010; Xiong et al., 2014). The elevated serum ferritin levels were observed in NSCLC and SCLC patients (Aleman et al., 2002; Kukulj et al., 2010; Yildirim et al., 2007), and in patients with cancer during radiotherapy (Koc et al., 2003). However, the expression of *FPN* in lung cancer cells has not been reported.

IRP1 is responsible for cytosolic iron concentrations and can post-transcriptionally regulate the expression of iron metabolism genes to maintain cellular iron homeostasis (Rouault, 2006). In cells with iron deficiency, IRP1 can bind to IRE element of ferritin mRNA, enhancing iron uptake and decreasing iron sequestration (Rouault, 2006). The tetracycline-inducible overexpression of *IRP1* or *IRP1*_*C437S*_ mutant results in misregulation of iron metabolism, highly active in IRE-binding and increased TfR1 levels in human H1299 lung cancer cells (Wang and Pantopoulos, 2002), but not altering the growth properties of the H1299 cells *invitro* (Wang and Pantopoulos, 2002). However, overexpression of *IRP1* or *IRP1*_*C437S*_ dramatically suppresses the growth of tumor xenografts in nude mice (Chen et al., 2007), providing a direct regulatory link between the IRE/IRP system and cancer. In A549 lung cancer cells, IRP2 can regulate the expression of *TfR1* and *ferritin* by changing its own gene expression, and thereby regulating iron metabolism (Cheng et al., 2014). Moreover, exhaled ferritin and superoxide dismutase (SOD) have recently been recognized to play a role in lung cancerogenesis and patients’ survival (Carpagnano et al., 2012), implying the possibility of using them as an outcome predictor of lung cancer.

Lipocalin-2 (LCN2), which is a member of the lipocalin family and functions to ligate ferric siderophore-like molecules, is involved in various cancers including lung cancer (Shiiba et al., 2013), breast cancer (Yang et al., 2013), ovarian cancer (Cho and Kim, 2009), colon cancer and pancreatic cancer (Mannelqvist et al., 2012). The elevated levels of LCN2 tightly associate with the malignance and metastasis of cancer cells (Leng et al., 2009; Yang et al., 2009). In lung cancer, LCN2 protein level increases significantly after X-ray irradiation (Shiiba et al., 2013), suggesting that overexpression of *LCN2* might contribute to radiation resistance in cancer cells (Shiiba et al., 2013).

### Breast cancer

Iron is strongly correlative to the risk of breast cancer through many aspects such as interaction with estrogen, disruption of lactoferrin, genetic variability in iron-related oxidative stress pathways, and abnormal expression of iron uptake or export genes (Torti and Torti, 2013).

The majority of breast cancers rely on supplies of estrogen to grow (Oh, 2002). Studies have revealed that estrogen contributes to breast cancer in multifactorial ways, and iron is suggested to interact with estrogen in two different ways (Torti and Torti, 2013). Generally, low level of iron may stimulate HIF1α activation and consequently promote angiogenesis, whereas high level of iron may increase oxidative stress (Torti and Torti, 2013). Recently, estrogen has been considered to contribute to iron homeostasis by regulating hepatic hepcidin expression directly through a functional estrogen response element (ERE) in the promoter region of hepcidin gene (Hou et al., 2012).

A unique correlation between iron and breast cancer is mediated by lactoferrin (Torti and Torti, 2013). Lactoferrin is a member of transferrin family and binds two ferric ions with high affinity (Torti and Torti, 2013; Ward et al., 2003). In human breast cancer cells, the exogenous lactoferrin, or adenovirus-mediated expression of lactoferrin can greatly inhibit cell proliferation (Duarte et al., 2011; Torti and Torti, 2013). In mice bearing EMT6 breast cancer, the injection of recombinant adenovirus containing lactoferrin can induce apoptosis and inhibit tumor growth (Wang et al., 2011), suggesting that lactoferrin has potential benefit in treating some breast cancers.

Several enzymes play critical roles in the formation and reduction of iron-generated ROS, including NAD(P)H:quinone oxidoreductase 1 (NQO1), nitric oxide (NO) synthase (NOS3) and heme oxygenase (HO) (Hong et al., 2007). NQO1 is important for breast carcinogenesis as it functions in the reduction of endogenous catechol estrogens (Hong et al., 2007). In animals, the suppression of *NQO1* increases estradiol-dependent tumor formation (Hong et al., 2007; Wyllie and Liehr, 1997). In mouse, the expression of *NQO1* is regulated by nuclear factor erythroid 2 related factor 2 (Nrf2) through consensus regulatory sequence known as the antioxidant response element (ARE) (Hong et al., 2007; Lin et al., 2011). Interestingly, Nrf2 can induce *ferritin-H* and *ferritin-L* gene expressions, leading to elevated cellular iron (Hong et al., 2007; Iwasaki et al., 2013). NOS3 generally functions to generate low levels of short-lived nitric oxide (NO) by converting L-arginine to citrulline in endothelial tissue (Hong et al., 2007). At low levels, NO acts as an antioxidant by scavenging ROS and can bind to iron to reduce redox cycling (Trachootham et al., 2008). The increased expression of *NOS3* has been detected in breast cancer, which is positively associated with estrogen and progesterone receptor status and negatively involved in histologic grade and lymph node status (Martin et al., 2000; Vakkala et al., 2000). HO also exhibits correlation with iron-related carcinogenesis as it catalyzes the rate-limiting step in heme degradation and provides cellular protection against oxidative stresses (Choi and Alam, 1996; Stocker, 1990).

The abnormal expression of iron homeostasis related genes are also widely examined in breast cancers. In human breast cancer MCF-7 cells, the expression of iron transporter genes including *TfR1*, *DMT1* and *FPN* is altered (Jiang et al., 2010). Compared to normal breast cells, the expression of iron importer genes is increased, whereas the expression of *FPN* is decreased in breast cancer cells to satisfy their increased demands for iron (Jiang et al., 2010). Interestingly, perturbations in ferritin levels are associated with breast cancer progression toward a more advanced malignant phenotype (Shpyleva et al., 2011). For instance, in human breast cancer cell lines with an epithelial phenotype, such as MCF-7, MDA-MB-361, T-47D and HCC70 cells, the expression of *ferritin-H*, *ferritin-L*, *Tf*, *TfR1*, *IRP1* and *IRP2* is decreased (Shpyleva et al., 2011). In contrast, the expression of these genes is commonly elevated in cells with an aggressive mesenchymal phenotype, such as Hs-578T, BT-549 and MDA-MB-231 cells (Shpyleva et al., 2011).

Additionally, LCN2 is a novel regulator of angiogenesis in human breast cancer (Yang et al., 2013). LCN2 functions to protect matrix metalloproteinase-9 (MMP-9) against degradation, which further enhances its enzymatic activity and facilitates angiogenesis and tumor growth (Fernandez et al., 2005). Overexpression of *LCN2* in MCF7 breast cancer cells increases the expression of vascular endothelial growth factor (*VEGF*), a key angiogenic activator (Yang et al., 2013). The LCN2-induced VEGF is mediated through HIF1α and that LCN2 regulates HIF1α through extracellular signal-regulated kinase (Erk) (Yang et al., 2013). However, some studies reported that inhibition of LCN2 results in breast tumorigenesis in two different mouse models (Berger et al., 2010; Leng et al., 2011).

### Prostate cancer

Prostate cancer is one of the most commonly diagnosed malignancies in men (Al Robaian et al., 2014). Iron also exhibits relevance to prostate cancer, including *FPN* level reduction (Chen et al., 2014), redox-sensitive transcription factor (NF-*κ*B) activation (Ornstein and Zacharski, 2007), the HIF1α-dependent pathway activation (Tsui et al., 2013), the association between heme iron intake and prostate cancer risk (Jakszyn et al., 2012), and high level of β2-microglobulin (β2-M) in patients with prostate cancer (Josson et al., 2013).

In prostate tumors, the Fpn protein level is significantly reduced by comparison with adjacent tissues, indicating a crucial role of Fpn in prostate tumor growth through controlling iron concentration (Chen et al., 2014). Conversely, inhibition of myeloid zinc-finger 1 (MZF-1) expression, an oncogene or a tumor suppressor, can lead to decreased Fpn level, enhancing tumor cell growth (Chen et al., 2014).

Treatment of the human prostate cancer cell line PC-3 with iron in the form of ferric nitrilotriacetate (FeNTA), but in the absence of added transferrin, results in stimulation of intracellular reactive oxygen intermediates (ROI) production, NF-*κ*B activation and increasing urokinase-type plasminogen activator (uPA) expression (Ornstein and Zacharski, 2007). These results imply that non-transferrin-bound iron (NTBI) may indirectly promote prostate cancer growth and metastasis (Ornstein and Zacharski, 2007). In PC-3 and LNCaP human prostate carcinoma cells, hypoxia dysregulates the expressions of lactate dehydrogenase A (*LDHA*), fatty acid synthase (*FASN*) and mitochondrial aconitase (*mACON*) genes (Tsui et al., 2013). In which, the hypoxia-induced *mACON* gene expression is via the HIF1α-dependent and iron-dependent pathways (Tsui et al., 2013).

Heme iron can promote endogenous production of NOCs (nitrosocompounds) and catalyze free radical formation, leading to oxidative cell damage (Jakszyn et al., 2012). The endogenous and exogenous dietary nitrosamines and heme iron intake have potential effect on prostate cancer risk (Jakszyn et al., 2012). Prostate cancer patients with bone metastasis exhibit elevated expression of β2-microglobulin (β2-M), which is a cell membrane protein (Josson et al., 2013). Previous studies have demonstrated that β2-M interacts with hemochromatosis protein (HFE), preventing excessive iron uptake (Josson et al., 2013). The β2-M/HFE complex is required for cancer development and bone metastasis (Josson et al., 2013). Genetic deletion of β2-M or HFE in prostate cancer cells exhibits sensitivity to radiation *invitro* and *in vivo* (Josson et al., 2013).

### Colorectal cancer

Studies have demonstrated that iron confers an increased risk for colorectal cancer (CRC). Commonly, increased cellular iron exposure is associated with CRC risk (Pusatcioglu et al., 2014). Hepcidin, a peptide hormone synthesized mainly in the liver, functions to inhibit iron transport by binding to Fpn (Rossi, 2005). Inappropriately elevated serum hepcidin may reduce duodenal iron absorption, which further increases colonic adenocarcinoma iron exposure in CRC patients (Pusatcioglu et al., 2014). Hereditary hemochromatosis, a genetic disorder of iron overload, generally harbors inappropriately elevated intestinal iron absorption (Emanuele et al., 2014). In HFE-associated hereditary hemochromatosis, mutations in the *HFE* gene disrupt the synthesis of hepcidin (Osborne et al., 2010). Decreased hepcidin levels result in increased release of iron from intestinal cells and macrophages, elevating plasma transferrin saturation and causing deposition of iron in the liver and other tissues (Osborne et al., 2010). *HFE* C282Y homozygotes have twice the risk of colorectal and breast cancer relative to those individuals without the C282Y variant hepcidin (Osborne et al., 2010).

The *Apc* (adenomatous polyposis coli) gene is the most commonly mutated tumor suppressor gene in sporadic colorectal cancer (Armaghany et al., 2012). After *Apc* deletion, TfR1 and DMT1 protein levels are rapidly induced (Radulescu et al., 2012). Conversely, restoration of APC reduces cellular iron due to repression of these two proteins (Radulescu et al., 2012). Deficiency of luminal iron significantly suppresses murine intestinal tumorigenesis, whereas increased luminal iron strongly promotes tumorigenesis (Radulescu et al., 2012). These results definitively delineate iron as a potent modifier of intestinal tumorigenesis (Radulescu et al., 2012).

Cellular iron uptake proteins such as DMT1 and TfR1 are up-regulated in CRC, whereas iron export proteins such as Fpn and hephaestin (HEPH) are down-regulated (Xue and Shah, 2013). In mouse models of colon cancer, iron-enriched diets increase the development of colon tumors (Tammariello and Milner, 2010). Moreover, hypoxia-inducible factor 2α (HIF2α) activation can promote colorectal cancer progression by dysregulating iron (Xue et al., 2012).

### Hepatocellular cancer

The liver is the most frequently affected organ by iron overload because iron is mainly stored in hepatocytes (MacKenzie et al., 2008). Hepatocellular carcinoma (HCC) commonly develops in patients with underlying hereditary hemochromatosis (HH) (Tan et al., 2009). Patients with HCC generally contain elevated iron in their livers, indicating the critical role of iron in stimulation of carcinogenesis (Kowdley, 2004). The wild-type HFE protein can form a complex with TfR, and two HFE mutations (C282T and H63A), and it also has been found to increase the affinity to the TfR (Beckman et al., 2000).

By using immunohistochemistry, the expression of *TfR1* and *TfR2* is significantly higher in human HCC tissues than in adjacent non-tumor tissues (Sakurai et al., 2014), suggesting that *TfR1* and *TfR2* are expressed in response to iron deficiency during liver carcinogenesis (Sakurai et al., 2014). Interestingly, in tumorous tissues from 24 HCC patients with chronic HBV infection, iron staining assay result by Perls’ Prussian blue was negative, whereas excess iron deposits were present in 17 of the 24 adjacent non-tumorous liver tissues (Tan et al., 2009). The expression of iron-regulatory genes, including *hepcidin*, *TfR2*, *Tf* and *IRP1* were significantly down-regulated in the tumorous tissues in comparison to the adjacent non-tumorous liver tissues (Tan et al., 2009). The proteomic and genomic evidences indicated that ferritin-L was suppressed in HCC (Park et al., 2002). The suppressed level of ferritin-L in HCC may be resulted from modification of IRPs, which may tightly bind to the IRE element of a target gene, thereby blocking *de novo* translation of ferritin-L (Park et al., 2002).

### Pancreatic cancer

Pancreatic cancer is one of the most fatal cancers in adult men and women (Bracci, 2012). Obesity is a modifiable risk factors associated with increased risk of pancreatic cancer (Bracci, 2012). Evidence suggests that obesity is associated with higher hemoglobin and ferritin concentrations (Hamalainen et al., 2012). Ferritin is detected at higher levels in the sera of many patients with pancreatic cancer (Kalousova et al., 2012). Additionally, labile iron has been implicated in both the pathogenesis and treatment of pancreatic cancer (Moser et al., 2013). In human pancreatic cancer cell line MIA PaCa-2, pharmacological ascorbate and irradiation have been shown to increase the labile iron in tumor homogenates (Moser et al., 2013). Heme-iron is associated with increased pancreatic cancer risk in female smokers (Molina-Montes et al., 2012).

An elevated expression of *LCN2* has been observed in pancreatic cancer (Leung et al., 2012). In two pancreatic ductal adenocarcinoma cell lines (BxPC3 and HPAF-II), the downregulation of *LCN2* significantly reduces attachment, invasion and tumour growth *in vivo*, but not proliferation or motility (Leung et al., 2012). In contrast, the overexpression of *LCN2* in PANC1 cells significantly increases invasion, attachment and tumor growth (Leung et al., 2012). LCN2 promotes the expression of *VEGF* and *HIF1α*, which contributes to the enhanced vascularity (Leung et al., 2012). These results indicate that LCN2 is critical for the malignant progression of pancreatic ductal carcinoma.

### Haematological malignancies

Haematological cancers include various types of blood cancers and related diseases such as acute and chronic leukaemias, myeloproliferative disorders, myelodysplastic syndromes and aplastic anemia (Koreth and Antin, 2010). Haematologic disorders commonly associate with iron overload due to increased iron absorption and hepcidin suppression (Koreth and Antin, 2010).

Patients with multiple myeloma (MM) have increased serum hepcidin, which inversely correlates with hemoglobin, suggesting that hepcidin contributes to MM-related anemia (Maes et al., 2010). Further studies revealed that hepcidin is induced by increased bone morphogenetic protein 2 (BMP2) (Maes et al., 2010). Hepcidin is upregulated by interleukin-6 (IL-6) in Hodgkin’s lymphoma (HL) patients in comparison to controls (Hohaus et al., 2010). Elevated levels of hepcidin in HL correlate with iron restriction and contribute to anemia (Hohaus et al., 2010). Additionally, hepcidin levels show a positive correlation with ferritin and an inverse correlation to iron and iron-binding capacity (Hohaus et al., 2010).

Two TfR1 antibodies, namely, 3TF12 and 3GH7, have been identified to exhibit abilities against tumors whose proliferation relies on high levels of TfR1 and iron uptake, such as acute lymphoid and myeloid leukemias (Crepin et al., 2010). Chelation of intracellular iron with the antifungal agent ciclopirox olamine (CPX) induces cell death in leukemia and myeloma cells (Eberhard et al., 2009).

## Iron and signal pathways in cancer

**Figure 4:**
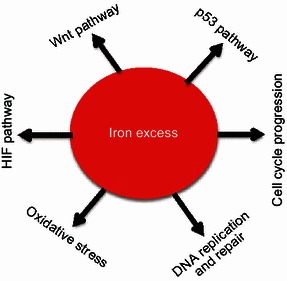
**Signal pathways in cancers caused by iron excess**. A variety of signal pathways are activated in multiple cancer types. These pathways mainly include p53 pathway, Wnt pathway, HIF pathway, DNA replication and repair pathway, cyclins and cell cycle regulation, and oxidative stress pathways

A variety of signal pathways are activated in different types of cancer cells, such as p53 pathway, Wnt pathway, HIF pathway, DNA replication and repair pathway, cyclins and cell cycle regulation, EGF pathway, AKT pathway and VEGF pathway. Interestingly, it has been found that iron is involved in most of these pathways (Fig. 4).

### Iron and p53

Iron status influences p53 activity and antioxidant response by modulating the expression of *MDM2* (mouse double minute gene 2) (Dongiovanni et al., 2010). In mouse hepatocytes and rat liver, iron status has been identified to regulate MDM2, which is associated with specific changes in p53 expression (Dongiovanni et al., 2010). MDM2 functions as a predominant negative regulator of p53, and it controls p53 activity and degradation through ubiquitination (Dongiovanni et al., 2010). Further, iron dependent regulation of MDM2/p53 has been confirmed in human monocytes (Dongiovanni et al., 2010). In which, the manipulation of iron pool leads to modulation of p53 target genes involved in the antioxidant response and apoptosis (Dongiovanni et al., 2010). Iron status influences p53 ubiquitination and degradation rate, and the MDM2 inhibitor nutlin increases p53 levels in iron-depleted cells (Dongiovanni et al., 2010).

Recently, iron metabolism has been revealed to regulate p53 signaling pathway through direct heme-p53 interaction and modulation of p53 localization, stability and function (Shen et al., 2014). Iron can directly affect the expression of *p53*, which is downregulated during iron excess (Shen et al., 2014). Iron polyporphyrin heme directly binds to p53 protein via the C-terminal CP motif (CACP) (Shen et al., 2014), which is a highly conserved DNA-binding domain (DBD) in eukaryotic p53 proteins (Shen et al., 2014). The binding leads to disruption of p53-DNA interactions, triggering both nuclear export and cytosolic degradation of p53 (Shen et al., 2014).

### Iron and Wnt pathways

There are three well-characterized Wnt signaling pathways, including Wnt/β-catenin pathway, noncanonical planar cell polarity (PCP) pathway and noncanonical Wnt/calcium pathway (von Maltzahn et al., 2012). Excessive signaling from the Wnt pathways has been implicated in the development of numerous types of cancers (MacDonald et al., 2009; von Maltzahn et al., 2012). Interestingly, previous studies have revealed a critical requirement of iron in Wnt signaling, and iron chelation serves as an effective mechanism to inhibit Wnt signaling in humans (Song et al., 2011). A series of acyl hydrazones can bind iron *in vitro* and in intact cells, and the chelating activity is required for abrogating Wnt signaling and blocking the growth of colorectal cancer cell lines with constitutive Wnt signaling (Song et al., 2011). In addition, multiple iron chelators, including desferrioxamine (DFO), deferasirox and ciclopirox olamine, function similarly to inhibit Wnt signaling and cell growth (Song et al., 2011). In an iron-induced mouse model of kidney cancer, an aberrant Wnt signalling was also examined (Torti and Torti, 2013). A soluble iron salt, namely, ferric nitrilotriacetate (FeNTA), can induce nephrotoxicity and kidney cancer in rats and mice models, suggesting that the activation of Wnt signalling may be a common pathway through which iron contributes to malignant progression (Torti and Torti, 2013).

Clearly, iron has important effects on Wnt signaling pathway, and iron mainly contributes to Wnt signaling pathway through the following two aspects: (1) it augments Wnt signalling in cells with aberrant APC or β-catenin, and (2) it downregulates E-cadherin which is essential for the induction and maintenance of polarized and differentiated epithelia in an APC-independent manner (Torti and Torti, 2013). These effects of iron on Wnt signalling may provide insight into the mechanism of iron exacerbating intestinal tumorigenesis, particularly in an APC mutation background (Torti and Torti, 2013).

### Iron and HIF pathways

Iron levels can also regulate hypoxia-inducible factor-α (HIFα) proteins, which are transcription factor subunits that are critical for the regulation of hypoxia response (Torti and Torti, 2013). There are three HIFα family members: HIF1α, HIF2α and HIF3α (Peyssonnaux et al., 2007). These subunits can form heterodimers with HIF1β (also known as ARNT), respectively, to function as the HIF transcription factors (Keith et al., 2012), which transcriptionally induce numerous genes responded to hypoxia, including vascular endothelial growth factor A (*VEGFA*), glucose transporter 1 (*GLUT1*; also known as *SLC2A1*), erythropoietin (*EPO*) and survivin (also known as *BIRC5*) (Torti and Torti, 2013). These HIFα subunits are posttranslationally regulated by iron-dependent prolyl-hydroxylases (PHD proteins) (Hewitson et al., 2003; Torti and Torti, 2013).

HIF protein activities are induced in many types of tumours, which are generally associated with tumour cell growth and progression (Torti and Torti, 2013). In cancer cells, HIF can induce the expression of a variety of iron metabolism genes, contributing to iron accumulation in cancer cells. For instance, HIF1α induces *TfR1* expression, resulting in increasing iron uptake (Lok and Ponka, 1999; Tacchini et al., 1999). HIF1α induces the expression of ceruloplasmin, which is a ferroxidase enzyme to oxidize iron, facilitating the loading of iron onto Tf (Mukhopadhyay et al., 2000). Moreover, HIF1α also induces *HO1* expression, leading to the degradation of heme into biliverdin, carbon monoxide and iron; as a result, which enables iron to be recycled (Lee et al., 1997). Additionally, HIF2α induces the expression of *FPN*, *DMT1* and *DCYTB* in enterocytes to promote systemic iron uptake (Peyssonnaux et al., 2007; Torti and Torti, 2013).

### Iron, DNA replication, repair and cell cycle progression

Defects in genes required for DNA replication and repair always cause genetic instability and are associated with multiple human cancer types (Preston et al., 2010). Interestingly, a variety of iron-containing proteins are involved in DNA replication and repair processes, such as DNA polymerases, DNA helicases, and the small subunit of ribonucleotide reductase (RNR) (Zhang, 2014). The eukaryotic DNA synthesis is generally performed via three conserved polymerases: Pol α, Pol δ and Pol ε (Miyabe et al., 2011). All of these DNA polymerases contain a Fe-S cluster in their active holoproteins (Zhang, 2014). DNA helicase and helicase-nuclease enzymes, including Xeroderma pigmentosum group D-complementing protein (XPD; also known as ERCC2), Fanconi anemia group J protein (FancJ), a member of the DEAD/DEAH subfamily of helicase ChlR1, regulator of telomere elongation helicase 1 (RTEL1) and DNA replication helicase 2 (Dna2), contain a conserved Fe-S cluster in their N-terminus, which is essential for helicase activities (Wu and Brosh, 2012). RNRs utilize radical chemistry to reduce ribonucleotides to synthesize deoxyribonucleotides (dNTPs), thereby generating the necessary precursors of DNA replication and repair (Zhang, 2014). Imbalanced dNTP pools are associated with increased DNA mutations, DNA strand breaks, and even cell death by enhancing misincorporation of nucleotides and by inhibiting DNA polymerase functions (Kumar et al., 2010; Zhang et al., 2014). The catalytic activity of RNR is dependent on a dinuclear iron site in the M2 subunit (RRM2) (Aye et al., 2014). Moreover, mammalian cells harbor a p53-inducible RRM2 subunit (p53R2, also known as RRM2B), which is induced in response to DNA damage (Torti and Torti, 2013). Interestingly, p53R2 is sensitive to iron chelation, thus it represents a target for iron chelation therapy in tumours with wild-type p53 (Torti and Torti, 2013).

Cells progress through the cell cycle unchecked may eventually result in malignant tumors (Tenga and Lazar, 2014). Iron is a major regulator of cell cycle by inhibiting either the formation or activities of the cyclin and cyclin-dependent kinase complexes (Zhang, 2014). Intracellular iron disruption by chelators leads to cell cycle arrest, particularly in G_1_ and S phases (Fu and Richardson, 2007; Siriwardana and Seligman, 2013). In many types of cancers such as sarcomas, colorectal and melanomas, cyclin D overproduction is observed (Burandt et al., 2014; Wang et al., 2014). Cyclin D1 associates with cyclin-dependent kinase 4 (CDK4) and CDK6 to regulate G_1_/S progression by phosphorylating retinoblastoma tumor suppressor protein (RB), which in turn releases the transcription factor E2F from RB (Torti and Torti, 2013).

### Iron and other pathways

Iron is also involved in AKT pathway and VEGF pathway in cancer cells. Iron oxide nanoparticles (Fe_2_O_3_ NPs) can mediate cytotoxicity via PI3K/AKT pathway, in which Fe_2_O_3_ NPs induce cellular damage and a natural flavonoid quercetin plays a protective role in Fe_2_O_3_ NPs induced cytotoxicity and apoptotic death (Sarkar and Sil, 2014). Iron-saturated lactoferrin stimulates cell cycle progression through PI3K/Akt pathway in MCF-7 cells (Lee et al., 2009). Vascular endothelial growth factor (VEGF) is a target of HIF, and its expression is induced in cells exposed to hypoxia (Forsythe et al., 1996). Thus, VEGF pathway is possibly involved in iron-mediated carcinogenesis.

## Therapeutic strategies based on iron deprivation

Elevated cellular iron may cause tumorigenesis, therefore decreasing iron level and targeting iron-containing proteins such as RNR have been developed as efficient strategies in chemotherapy. Numerous iron chelators, such as DFO, triapine, deferiprone, deferasirox and tachpyridine, have been preclinically or clinically used as anticancer agents (Torti and Torti, 2013). Of them, DFO and triapine are the most common iron chelators used in cancer therapy. DFO has antiproliferative effect both *invitro* and *invivo*, which is mediated by its depleting effect on intracellular iron pools (Dayani et al., 2004). However, the clinical efficiency of DFO is limited by its poor membrane permeability and a very short half-life in the bloodstream (Chaston and Richardson, 2003; Lovejoy and Richardson, 2003). Triapine exhibits wide-spectrum antitumor activity and cytotoxicity (Buss et al., 2003; Yu et al., 2006). Studies suggest that the cytotoxicity of triapine involved multiple mechanisms, such as by forming complexes with both Fe^2+^ and Fe^3+^, and by inhibiting both RRM2 and p53R2 (Enyedy et al., 2011; Zhang et al., 2014). The Fe^2+^-triapine can react with O_2_ to produce reactive oxygen species (ROS) (Richardson et al., 2009).

Moreover, some strategies that generate single-chain antibodies targeted towards TfR1 also have been developed (Crepin et al., 2010; Torti and Torti, 2013). TfR1 is mainly targeted in two different ways: (1) delivering therapeutic molecules into malignant cells, and (2) blocking the natural function of the receptor leading directly to cancer cell death (Daniels et al., 2012). Numerous types of anti-cancer drugs targeted TfR1 have been developed, such as a variety of anti-TfR antibodies (HB21, 454A12, B3/25, OKT9, 7D3, 7579 and 42/6) (Daniels et al., 2012), and multiple tumor-targeting ligands which are responsible for the delivery of numerous antitumour cytotoxics (TF-doxorubicin, TF-cisplatin, TF-chlorambucil, TF-ricin A chain and TF-diptheria toxin) (Torti and Torti, 2013). Some studies have obtained promising results for the treatment of a variety of cancers via cytotoxicity induced by the direct inhibition of TfR1 function by its monoclonal antibodies (Daniels et al., 2012). These tumor-targeting ligands can be delivered into cancer cells, causing cytotoxic effects including growth inhibition and/or induction of apoptosis in a variety of malignancies *in vitro* and *in vivo* (Daniels et al., 2012).

## Conclusion

Collectively, altered iron metabolism is a key hallmark of cancer. Iron diversely functions in tumor microenvironment, cancer initiation, progression and metastasis. A variety of signal pathways are activated by iron in cancer cells, and the expression of numerous iron metabolism genes is aberrant in malignant tumors, suggesting the fundamental roles of iron in developing cancer. Although great progresses have been made in the previous studies, the detailed understandings on the mechanisms of iron homeostasis regulation, iron-containing protein functions, and signal transduction involved in iron in cancer cells are still required to further explore.
